# Enhanced Electromechanical Property of Silicone Elastomer Composites Containing TiO_2_@SiO_2_ Core-Shell Nano-Architectures

**DOI:** 10.3390/polym13030368

**Published:** 2021-01-25

**Authors:** Shuyan Gao, Hang Zhao, Na Zhang, Jinbo Bai

**Affiliations:** 1 State Key Laboratory of Photon-Technology in Western China Energy, Institute of Photonics & Photon-Technology, Northwest University, Xi’an 710069, China; Gaoshuyann@126.com (S.G.); zhangna1@stumail.nwu.edu.cn (N.Z.); 2 Laboratoire de Mécanique des Sols, Structures et Matériaux, CNRS UMR 8579, Centrale-Supélec, Université Paris-Saclay, 8-10 rue Joliot Curie, 91190 Gif-sur-Yvette, France

**Keywords:** dielectric composites, electro-active polymers, dielectric elastomers, core-shell structure, electromechanical actuation

## Abstract

Dielectric elastomer (DE) is one type of promising field-activated electroactive polymer. However, its significant electromechanical actuated properties are always obtained under a giant electric voltage, which greatly restricts the potential applications of DE. In the present work, the well-constructed core-shell TiO_2_@SiO_2_ nanoparticles were fabricated by using the classical Stöber method. A series of TiO_2_@SiO_2_ nano-architectures-filled polydimethylsiloxane (PDMS) composites were prepared via solution blending and compression-molding procedures. Benefiting from the additional SiO_2_ shell, both the interfacial compatibility between fillers and matrix and core-shell interfacial interaction can be improved. The TiO_2_@SiO_2_/PDMS nanocomposites exhibit a significantly enhanced in-plane actuated strain of 6.08% under a low electric field of 30 V·μm^−1^ at 16 vol.% TiO_2_@SiO_2_ addition, which is 180% higher than that of neat PDMS. The experimental results reveal that the well-designed core-shell structure can play an important role in both improving the electromechanical actuated property and maintaining a good flexibility of DE composites. This research provides a promising approach for the design of the novel composites with advanced low-field actuated electromechanical property in next generation DE systems.

## 1. Introduction

Dielectric elastomer (DE) is one type of promising field-activated electroactive polymer (EAP), which shows a relatively large activated stress and strain when under an electric field [1,2,3,4]. Compared with electroactive ceramics (EACs), shape memory alloys (SMAs), electro-strictive materials [5,6], and other traditional driving materials, DE materials exhibit distinctive superiorities such as light-weight, large deformation, high energy-density, rapid response, and ease-processing, etc. [7,8]. All these features endow DE materials with a great application potential in bioinspired tunable optical lens [9], micro-optical submount [10]; energy generator from human motion [11] and sea wave [12], flexible robotic arm [13], software robot [14], electric-fish-inspired actuator [15], gene transfection driver [16], soft logic switch [17], etc.

The electromechanical actuation performance of dielectric elastomer actuators (DEAs) is critical to evaluate the energy conversion from electrical to mechanical energy. However, in order to obtain sufficient actuation stress and strain, an undesired huge electric voltage has to be applied to DEA materials, which severely hinders a majority of practical applications of DEAs [18]. From the perspective of material design, the electro-driving stress is mainly determined by both the relative dielectric constant (*ε_r_*) and breakdown strength (*E_b_*) of DE [19]. Moreover, the considerable electroactive strains are always obtained from DEs with low Young’s modulus (*Y*) values. Besides, mechanical strength is also considered as an important parameter in influencing the overall performance of DEAs [20].

Recently, several strategies have been widely used in regulating the functional characteristics of DEs. The first pathway is to effectively improve the *ε_r_* of DEs. For instance, filling conductive fillers according to the percolation effect (metallic and carbon materials, etc.) [21,22,23], high-*ε_r_* ceramic fillers based on mixing theory (e.g., BaTiO_3_, TiO_2_, CaCu_3_Ti_4_O_12_, etc.) [24,25,26], and certain organic dipoles (such as azobenzene) [27] into elastomeric matrix are frequently-adopted approaches. However, due to the consequent severely weakened dielectric strength, flexibility, and processability, these traditional strategies are always hard to meet the expectation of electro-active performance optimization. In addition, the trails of tuning the flexibility of DEs by incorporating plasticizers have been considered another effective approach in optimizing the electro-actuation property at low fields. The reported plasticizers such as dioctyl phthalate (DOP) and dimethyl silicone oil can cause DE materials to deform more easily in a certain electric field [28,29]. However, this method results in the decay of both *E_b_* and *ε_r_* of DEAs. In our previous study, a dimethyl silicone oil-plasticized BaTiO_3_/polydimethylsiloxane (BT/PDMS) DE composite was prepared with an improved electromechanical actuation sensitivity and strain through regulating both *ε_r_* and *Y* of DEs system cooperatively [30]. However, the problem of plasticizer effusion still needs to be solved in further studies.

Core-shell structure is a heterogeneous system, assembled by one material coated by another material with different features through chemical bonds or other interactions. The interfacial compatibility and interaction between core-material and matrix can be effectively regulated by core-shell structures. Thus, well-constructed core-shell fillers can be used to optimize the overall functionalities of composites. In recent years, the way of using core-shell fillers to compromise the mismatch between traditional high-*ε_r_* fillers and low-*ε_r_* matrix has been considered to be reasonable in hindering the decrease of *E_b_* of a composite. Consequently, it can improve the energy-storage properties of polymer-based film capacitors [31]. Compared with traditional polymer-based film capacitors, DE is much more sensitive to the filler-loading concentration. In order to preserve its electro-actuation ability, DE composites must possess high *ε_r_* and flexibility simultaneously. Thus, the filler-loading should be suppressed as low as possible. In fact, a well-designed core-shell particle with an additional buffer shell could endow composites with an additional heterogeneous interface, a stronger interfacial polarization, and a larger polarizability in comparison with a bulk core-particle. Consequently, it is possible to obtain DEs with an improved electro-actuation ability by employing core-shell structures as functional fillers. Yang et al. [32] prepared epoxy-functionalized silane@poly(catechol/polyamine)@BT core-shell nanoparticles. Due to its positive contribution to both dispersion and interfacial interaction, 13.4% actuation strain was obtained from the natural rubber-based composite filled with 50 phr fillers at 90 kV∙mm^−1^. Liu et al. [33] fabricated a TiO_2_@urea core-shell particle-loaded DE composite, from which an improved lateral actuation strain of 7.53% at 8.5 V·μm^−1^ was obtained.

Compared with general DE matrices such as polyurethane [34,35] and acrylic [36], due to its superior merits of low viscoelasticity, fast response time, ease-processing, and excellent biocompatibility [1], PDMS has been frequently adopted as DE matrix in recent reports [37,38]. In this study, the core-shell structured TiO_2_@SiO_2_ nanoparticles were synthesized based on the classical Stöber method [39]. A series of TiO_2_@SiO_2_/PDMS composites with various filler-loadings were prepared by solution-blending and compression-molding procedures. The typical multi-layered heterogeneous structure of core-shell-matrix with gradually-reduced *ε_r_* was built, in order to weaken the electrical mismatch in DE system as much as possible. Moreover, the SiO_2_-shell can improve the interfacial compatibility between TiO_2_ and PDMS matrix, and prevent the low-*ε_r_* weak-interaction interface from being formed in composites [40]. Simultaneously, the mechanical, dielectric, and electromechanical actuation behaviors of TiO_2_@SiO_2_/PDMS composites were carefully studied. The strategy of constructing core-shell structure as functional unit of DE provides a feasible route for design and preparation of DEAs in the next generation.

## 2. Experimental

### 2.1. Materials

Hydroxyl-terminated PDMS (3000 mPa·s) was purchased from Shandong Wancheng chemical company, Jinan, China. Nano TiO_2_ (anatase, spindle-shaped with a short and long diameter of 15 nm and 30 nm, respectively) and carbon black (CB) were purchased from Pioneer Nano Co., Ltd. (Nanjing, China). Dibutyltin dilaurate (AR), ethyl silicate (TEOS, AR), absolute ethanol (AR), and ammonia (25 wt.%, CP) were obtained from Kermel Chemical Company, Tianjin, China. Tetrahydrofuran (THF) was supplied by Sinopharm Chemical Reagent Company, Shanghai, China. All chemicals were used as received without further purification.

### 2.2. Synthesis of TiO_2_@SiO_2_ Nanoparticles

Typically, 1.44 g of TiO_2_ was dispersed into the mixture of ethanol (240 mL) and deionized water (54 mL) by ultrasonic treatment with 120 W for 30 min and magnetic stirring for another 30 min to obtain a homogenous suspension. Then, the concentrated ammonia solution (6 mL, 25 wt.%) was added into the mixture solution followed by a vigorous magnetic stirring for 30 min. The main function of ammonia solution was to provide alkaline environment for the reaction system. A total of 2.4 mL of TEOS was added dropwise into the above solution, and the reaction was allowed to proceed for 12 h at room temperature under continuous magnetic stirring. The well-reacted suspension was aged at room temperature for 24 h, then washed by ethanol and deionized water alternately several times. Afterwards, the target product was obtained after drying at 60 °C for 12 h.

### 2.3. Fabrication of TiO_2_@SiO_2_/Polydimethylsiloxane (PDMS) Composites

Firstly, a certain amount of TiO_2_@SiO_2_ was dispersed into ethanol with ultrasonic treatment for 30 min. Meanwhile, PDMS was dissolved into THF with magnetic stirring until a homogenous solution was obtained. The two suspensions were blended and vigorously stirred at 40 °C for 5 h, followed by 50 °C for another 2 h to remove the solvent in a vacuum oven. Then the TEOS and dibutyl tin dilaurate, with the mass ratio of 20:3, were added and dispersed into the above mixture by stirring clockwise for 1 min. The obtained viscous mixture was casted into a lab-fabricated stainless steel mold and vulcanized at room temperature for 12 h under a pressure of 15 MPa. Typically, the relevant preparation procedures of core-shell TiO_2_@SiO_2_ particles and TiO_2_@SiO_2_/PDMS composites are demonstrated in Figure S1. The neat PDMS and TiO_2_/PDMS composites were also fabricated by the above-mentioned procedure, in order to systemically study the relevant contribution of core-shell fillers.

### 2.4. Characterization and Measurements

The morphologies of the TiO_2_@SiO_2_/PDMS composites, TiO_2_ and TiO_2_@SiO_2_ nanoparticles were observed by a field emission scanning electron microscope (SEM, Gemini SEM 300, Oberkochen, Germany). The microstructure of core-shell nanoparticles was observed by high-resolution transmission electron microscopy (TEM, G2 F20 S-TWIN, OR, USA). The crystal structure of all samples was investigated by using X-ray diffraction (XRD) (DanDong HaoYuan DX-2700, Liaoning, China) using Cu Kα radiation (λ = 1.54 Å) at the 2θ range from 20° to 80°. The X-ray photoelectron spectroscopy (XPS) analysis was determined on a PHI-5400 electron spectrometer (ULVCA-PHI, Chigasaki, Japan) with Mg Ka as the X-ray source. The surface chemistry of the samples was measured by using a Fourier transform infrared (FT-IR) spectrometer (PerkinElmer, Paragon1000, Waltham, MA, USA). The mechanical properties of the samples were tested by using a EZ Test (EZ-LX, Shimadzu Corporation, Kyoto, Japan) machine at a crosshead speed of 10 mm/min, and the test sample is prepared in dumbbell shape according to GBT-528 IV. The elastic modulus of the sample was determined by calculating the slope of the tensile stress-strain curve in the linear region. The dielectric properties of the composites were measured on a precision impedance analyzer (4294A, Agilent Technologies, Santa Clara, CA, USA) over the frequency ranging from 10^3^ Hz to 10^6^ Hz at room temperature. Silver paste was evenly coated onto samples surfaces before measuring in order to remove the contact resistance. The electromechanical performance and dielectric strength were determined by using a direct current (DC) ultra-high voltage source (CS9920B, Nanjing, China) under a prestrain-free experimental condition, and a multi-directional real-time image-recording system. Two concentric circular CB electrodes were coated on opposite sides of the elastomer film, as shown in Figure S1. Copper wires were fixed onto the patterned electrode in order to conduct between DE film with an external electric field (see Figure S1).

## 3. Results and Discussion

### 3.1. Morphology and Characterization of TiO_2_@SiO_2_ Nanoparticles

**Figure 1:**
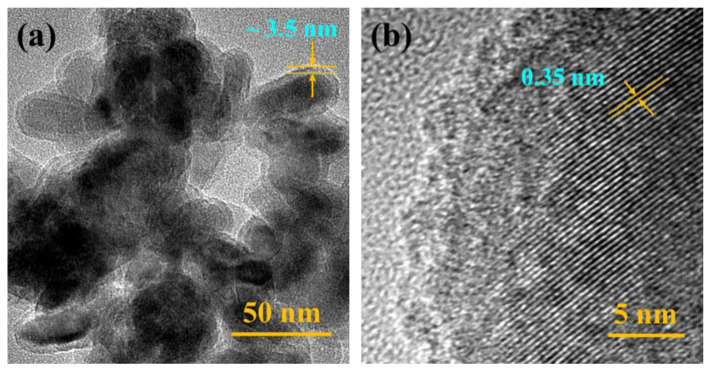
Transmission electron microscopy (TEM) image of (**a**) TiO_2_@SiO_2_ nanoparticles and (**b**) presents a high resolution TEM image of TiO_2_@SiO_2_ nanoparticles.

The morphology of TiO_2_ and TiO_2_@SiO_2_ nanoparticles are characterized by SEM and TEM. As shown in Figure 1, the successfully prepared core-shell structure of TiO_2_@SiO_2_ nanoparticles can be clearly observed in TEM images. The interplanar spacing is about 0.35 nm, which corresponds to the (101) crystal plane of TiO_2_ (Anatase, PDF#21-1272), as displayed in Figure 1b. The spindle-shaped TiO_2_ nanoparticles are completely encapsulated by a SiO_2_ shell with a uniform thickness of about 3.5 nm, and neat SiO_2_ nanoparticles cannot be observed (see Figure 1a). Both as-received TiO_2_ and as-prepared TiO_2_@SiO_2_ nanoparticles possess relatively uniform shapes and sizes (see Figure S2a,b). In addition, the SEM-EDX is presented in Figure S2c, which indicates that the as-prepared TiO_2_@SiO_2_ nanoparticles are mainly composed of three elements: Ti, Si, and O. It further demonstrates that the TiO_2_ nanoparticles are coated with SiO_2_ shell-layer.

**Figure 2:**
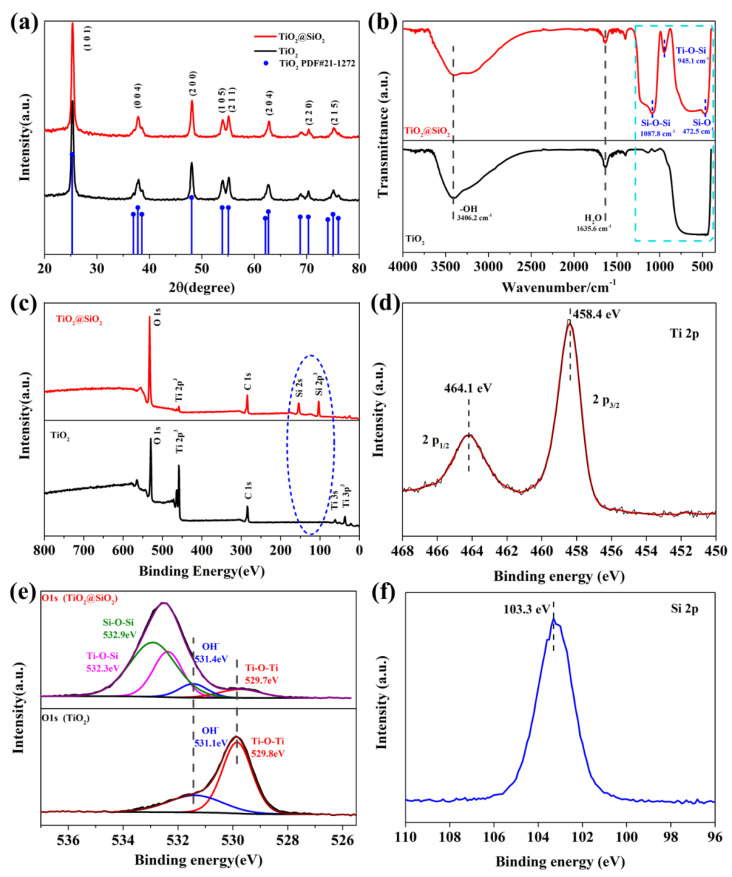
(**a**) X-ray diffraction (XRD) patterns, (**b**) Fourier transform infrared (FT-IR) spectra and (**c**) X-ray photoelectron spectroscopy (XPS) results of TiO_2_ and TiO_2_@SiO_2_ nanoparticles; (**d**) Ti 2p XPS spectrum of TiO_2_@SiO_2_ nanoparticles, (**e**) high-resolution signals of O 1s elements of TiO_2_ and TiO_2_@SiO_2_ nanoparticles, (**f**) Si 2p XPS spectrum of TiO_2_@SiO_2_ nanoparticles.

In order to further verify the crystal structure, X-ray diffraction (XRD) characterization was performed to TiO_2_@SiO_2_ nanoparticles. As shown in Figure 2a, there is no obvious new phase and changes in crystal are found in XRD patterns, which confirm that SiO_2_ shell has an amorphous structure. Figure 2b displays the FT-IR spectra of TiO_2_ and TiO_2_@SiO_2_ nanoparticles. The wide absorption band at 400–600 cm^−1^ on both curves represents the stretching patterns of Ti-O and Ti-O-Ti bonds, corresponding to core-TiO_2_ [41,42]. Moreover, there are obvious absorption bands at 1087.8 cm^−1^ and 472.5 cm^−1^ in TiO_2_@SiO_2_ spectrum, which correspond to the stretching mode of Si-O-Si asymmetric bond and the bending vibration of Si-O bond, respectively [42,43,44]. This result confirms the existence of silica. It is noteworthy that a weak peak can be found at 945.1 cm^−1^, which represents the vibration of Ti-O-Si bond [45,46]. It indicates that SiO_2_ shell is connected to the surface of TiO_2_ through chemical bonds.

The X-ray photoelectron spectroscopy patterns of TiO_2_ and TiO_2_@SiO_2_ are shown in Figure 2c–f, which further confirms the composition of shell layer. Figure 2c presents the fully scanned spectra in the range of 0–800 eV. The XPS pattern of the TiO_2_@SiO_2_ sample indicates the presence of Ti, Si, O elements (C 1s is the calibration peak) in core-shell structure, which is in accordance with the results of SEM-EDX. As shown in Figure 2d, two characteristic peaks at 458.4 eV and 464.1 eV are corresponded to Ti 2P_3/2_ and Ti 2P_1/2_ binding energies, respectively [47]. Moreover, the curve fitting of O 1s peaks of two samples shown in Figure 2e. The peaks at 529.7 eV, 531.4 eV, 532.3 eV, and 532.9 eV indicate the functional groups of Ti-O-Ti, hydroxyl (OH^−^), Ti-O-Si, and Si-O-Si, respectively [42,45,46]. The peaks appearing at the binding energy of 103.3 eV can be assigned to the 2p of Si, indicating the formation of SiO_2_ shell (Figure 2f). In summary, the XPS result further demonstrates that the typical TiO_2_@SiO_2_ structures are successfully synthesized and the interface between TiO_2_-core and SiO_2_-shell are tightly connected by chemical bonding.

### 3.2. Morphology of TiO_2_@SiO_2_/PDMS Composites

**Figure 3:**
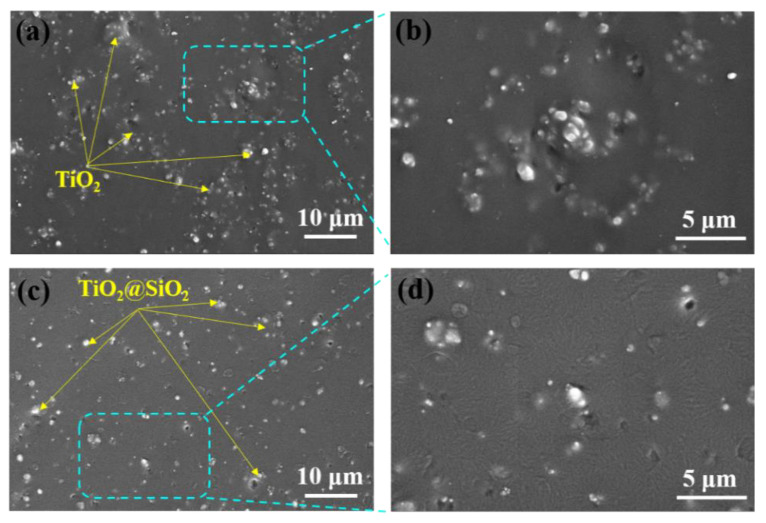
Cross-section SEM images of composites with filler content of 4 vol.%, (**a**,**b**) TiO_2_/polydimethylsiloxane (PDMS) composites and (**c**,**d**) TiO_2_@SiO_2_/PDMS composites.

The SEM images of fractured-surface TiO_2_/PDMS and TiO_2_@SiO_2_/PDMS composites are shown in Figure 3. Compared with TiO_2_/PDMS composites, TiO_2_@SiO_2_ nanoparticles are well-dispersed in PDMS matrix without obvious aggregation, as shown in Figure 3a,c. This is attributed to the large difference in surface energy between fillers and polymer matrix. Moreover, as displayed in Figure 3b,d, the interface between TiO_2_@SiO_2_ and PDMS matrix is more blurry than TiO_2_/PDMS interface, which indicates the additional SiO_2_-shell endows TiO_2_@SiO_2_/PDMS composites with a better interfacial compatibility and adhesion.

### 3.3. Functional Properties of TiO_2_@SiO_2_/PDMS Composites

**Figure 4:**
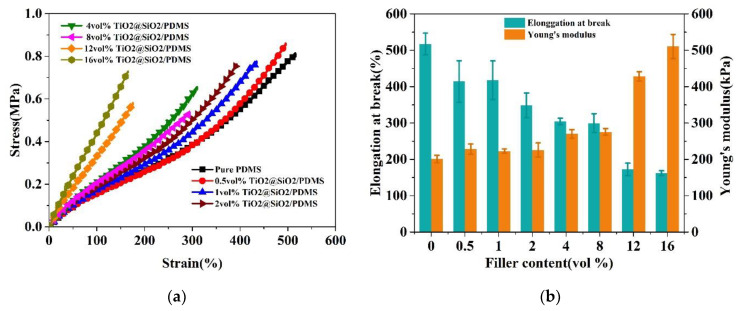
(**a**) Stress-strain curves, (**b**) elongation at break and Young’s modulus of PDMS composites filled with different contents of TiO_2_@SiO_2_ nanoparticles.

Figure 4a and Figure S3 illustrate the typical stress-strain behaviors of TiO_2_@SiO_2_/PDMS composites and TiO_2_/PDMS composites with different loadings, respectively. In order to ensure uniaxial stretch test reliability, five testing samples were prepared for each composite composition. It is observed that the stress-strain behavior of a composite transfers from non-linear to quasi-linear, when TiO_2_@SiO_2_ nanoparticles’ loading fraction increases. The main reason for this transition is that a growing number of functional fillers worked as physical crosslinking points and interfacial reinforcement, which hinders the movement of flexible elastomer macromolecules. In fact, the increase of TiO_2_@SiO_2_ loading content is able to reduce the overall flexibility of the PDMS-based DE composites, which makes the relevant mechanical breakdown occur more easily in the linear stress-strain region.

As exhibited in Figure 4b, the elongation at break value of TiO_2_@SiO_2_/PDMS composites decreases with an increased filler loading. Compared with PDMS matrix, the elongation at break of composites loaded with 12 vol.% and 16 vol.% decreased by 66% and 69%, respectively. It can be explained as follows: when the composites are applied to a uniaxial stretching, a majority of stress can be transferred from crosslinked PDMS macromolecular chains to the interface between TiO_2_@SiO_2_ nanoparticles and PDMS matrix, resulting in uneven stress on each part of TiO_2_@SiO_2_/PDMS composites. The number of force-bearing points inside composites grows fast with the increasing loading fraction, which results in a significant decay in the elongation at break of TiO_2_@SiO_2_/PDMS composites. The neat PDMS exhibits a relatively low Young’s modulus of 201 kPa, which is flexible enough to be employed as DEA matrix. As mentioned before, both the movement-impeded PDMS macromolecular chain and internal interfacial reinforcement are mainly responsible for the enhancement of Young’s modulus of PDMS-based composites with growing TiO_2_@SiO_2_ loading fractions. It is observed that when the TiO_2_@SiO_2_ loading is higher than 8 vol%, the corresponding *Y* value of the composite increased significantly. Specifically, the *Y* values of the composite incorporated with 12 vol.% and 16 vol.% TiO_2_@SiO_2_ are 428 kPa and 511 kPa, which increased by 113% and 154% in comparison with neat PDMS, respectively.

**Figure 5:**
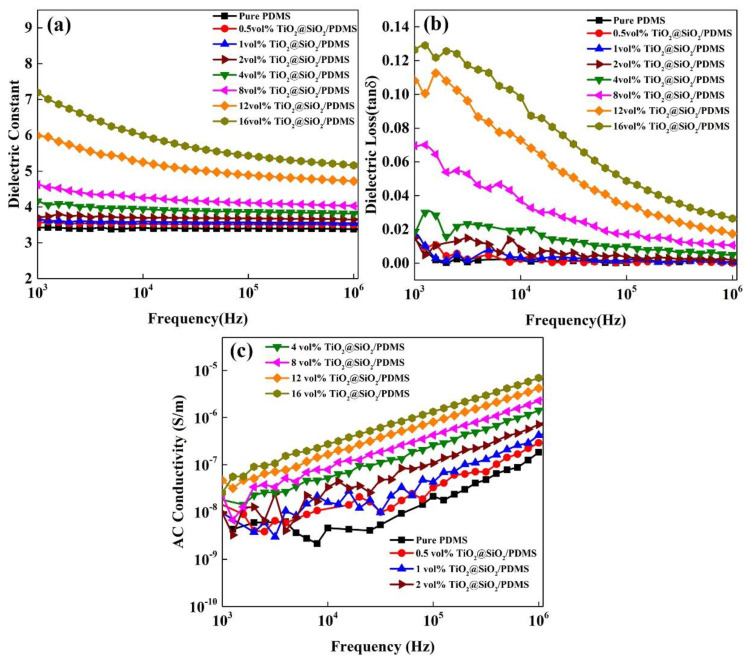
Frequency dependence of (**a**) dielectric constant, (**b**) dielectric loss, and (**c**) alternating current (AC) conductivity of TiO_2_@SiO_2_/PDMS composites.

Figure 5 shows the dielectric performance curves of the TiO_2_@SiO_2_/PDMS composites with different loading fractions at room temperature. As depicted in Figure 5a and Figure S4a, the dielectric constants of both TiO_2_/PDMS and TiO_2_@SiO_2_/PDMS composites are basically independent to the AC-frequency when the filler loading is below 4 vol.%, which is ascribed to the dielectric polarization being strongly influenced by the filler incorporating fraction [48]. However, when the filler loading is beyond 4 vol.%, the *ε_r_* value of TiO_2_@SiO_2_/PDMS composites exhibits a significant negative frequency-dependence. The decay rate is fast in the low-frequency range, and slows down with an increasing frequency. This typical frequency-dependence behavior is mainly derived from the combination effect of multi-type polarization which exists in TiO_2_@SiO_2_/PDMS composites, such as interfacial polarization of heterogeneous interfaces [49], and dipole orientation polarization of fillers and polymer matrix. When an external electric field applied to the composite, free charges could accumulate at the interfaces between core (TiO_2_)-shell (SiO_2_), shell (SiO_2_)-matrix (PDMS), and also the crystal defects in PDMS matrix. It gives rise to an uneven space charge distribution (i.e., resulting in a macroscopic dipole moment), thereby being responsible for the enhancement of both overall polarization and *ε_r_* values. This is why the *ε_r_* value of composites is high at low frequencies (~10^3^ Hz). On the other hand, when the frequency rises to 10^3^–10^7^ Hz, the dipole orientation becomes the main polarization mode, where the dipole orientating speed could not keep up with the growing AC-field changing frequency. Therefore, a weakened overall polarization and relatively stable *ε_r_* value of the composites are obtained. In addition, the *ε_r_* value of composites augments with an increasing TiO_2_@SiO_2_ nanoparticles loading fraction, which is ascribed to the enhanced polarization. Notably, the *ε_r_* value of the composite filled with 16 vol.% TiO_2_@SiO_2_ is 8.91 at 100 Hz, which is about 316% higher than that of PDMS. This value is also higher than that of Ag@SiO_2_/PDMS reported by Xiong et al. [50].

As illustrated in Figure 5b, the dielectric loss (tan δ) of TiO_2_@SiO_2_/PDMS composites shows a similar frequency-dependent behavior with *ε_r_* values at room temperature. The polarization loss is the main loss mode in the TiO_2_@SiO_2_/PDMS composites, whose value is directly dependent on the matching degree between AC electric field frequency and dipole motion. In the high frequency range, due to the dipole orientation being unable to keep up with the change of AC electric field, the weakened orientation polarization is responsible for the reduced tan δ values. In fact, although the dielectric loss of TiO_2_@SiO_2_/PDMS composites grows with an increasing filler loading, the overall values in the whole testing frequency range are well kept at a very low level (tan δ < 0.13). Compared with TiO_2_/PDMS counterpart, the core-shell filled composites display slightly larger dielectric loss values, indicating that the additional core-shell heterogeneous interface would not give rise to a significantly enhanced dielectric loss (see Figure S4b).

As exhibited in Figure 5c, the composite filled with more TiO_2_@SiO_2_ particles shows a higher AC conductivity (*σ_AC_*) in the whole testing frequency range. In a high frequency range, electrons are able to be sufficiently excited and jump among neighboring conductive clusters, which leads to the linearly grown *σ_AC_* values of TiO_2_@SiO_2_/PDMS composites with the increasing testing frequency.

**Figure 6:**
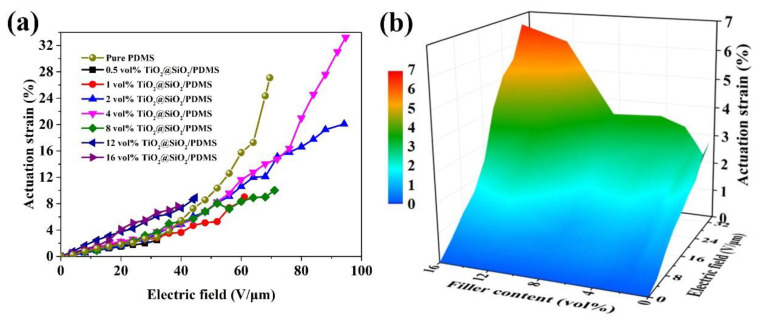
(**a**) Dependence of the actuated strain of TiO_2_@SiO_2_/PDMS films with different TiO_2_@SiO_2_ volume fractions on the applied electric field; (**b**) the actuation strain of composites at the electric field from 0 to 30 V∙μm^−1^.

The electro-actuated strain of TiO_2_@SiO_2_/PDMS composites with different loading fractions are shown in Figure 6a. The corresponding operating principle of dielectric elastomer actuator is shown in Figure S5. When an augmenting electric field was applied, the actuated strain of composite films gradually increases. Moreover, the actuated strains of the composites with higher TiO_2_@SiO_2_ loading fraction are larger than those of the lower TiO_2_@SiO_2_ incorporated composites at a certain electric field. This could be attributed to the additional polarization provided by the TiO_2_@SiO_2_ core-shell interface. The actuated strain of the composite filled with 4 vol.% TiO_2_@SiO_2_ nanoparticles is up to 33.2% at 93 V·μm^−1^, which is higher than reported in the literature [32]. However, such a high electric field would make most of potential applications of DEs become impractical. Consequently, the contribution to the low-field actuated performance of DE composites made by synthesized core-shell architecture is more worthy to be investigated. When the electric field is reduced below 30 V·μm^−1^, the composite filled with 16 vol.% TiO_2_@SiO_2_ displayed the largest actuated strains. As demonstrated in Figure 6b, the actuated strain can reach to 6.08% at 30 V·μm^−1^, which is about 180% higher than that of neat PDMS (strain = 2.17%).

Notably, compared with TiO_2_ filled system, at the same loading, the TiO_2_@SiO_2_ filling volume is the sum of TiO_2_ volume and relatively low-*ε_r_* SiO_2_ volume. However, unlike the TiO_2_/SiO_2_ random mixing state, the well-designed TiO_2_@SiO_2_ core-shell structure could endow the composites with significantly different properties. Therefore, both the additional heterogeneous core-shell interfacial interaction and improved shell-matrix interfacial compatibility would be mainly responsible for the optimized electro-actuated performance of TiO_2_@SiO_2_/PDMS DE composites.

## 4. Conclusions

In summary, the homogeneous core-shell TiO_2_@SiO_2_ nanoparticles were fabricated by using the classical Stöber method. A series of TiO_2_@SiO_2_/PDMS composites were prepared by solution blending and compression-molding procedures. The experimental results indicated that the addition of SiO_2_ shell can improve the interfacial compatibility between TiO_2_@SiO_2_ nanoparticles and PDMS matrix. The TiO_2_@SiO_2_/PDMS composite filled with 16 vol.% TiO_2_@SiO_2_ exhibited both significantly enhanced dielectric constant (8.91 at 100 Hz) and actuated strain (6.08% at 30 V∙μm^−1^), which are about 3.16 times and 1.80 times higher than that of pure PDMS, respectively. Moreover, although the Young’s modulus of the composites slightly enhanced with the growing TiO_2_@SiO_2_ loading fraction, the composites still showed good flexibility. As a result, the strategy of constructing core-shell structure is reasonable to elevate the polarization efficiency of functional fillers, which offers a promising route for achieving novel DE composites with excellent low electric-field actuated electromechanical properties.

## Data Availability

The data presented in the study are available on request from the corresponding author.

## Supplementary Materials

The following are available online at https://www.mdpi.com/2073-4360/13/3/368/s1, Figure S1: Schematic diagram of the preparation procedure of TiO_2_@SiO_2_ core-shell nanoparticles and composites, Figure S2: Scanning electron microscopy (SEM) images of (a) TiO_2_ and (b) TiO_2_@SiO_2_ nanoparticles; (c) energy-dispersive X-ray spectroscopy (EDS) image of TiO_2_@SiO_2_ nanoparticles, Figure S3: Stress-strain curves of TiO_2_/polydimethylsiloxane (PDMS) composites, Figure S4: Frequency dependence of (a) dielectric constant, (b) dielectric loss in TiO_2_/PDMS composites, Figure S5: Operating principle of dielectric elastomer actuator.
