# Supplementary material for: Enhanced Electromechanical Property of Silicone Elastomer Composites Containing TiO2@SiO2 Core-Shell Nano-Architectures
Source: Polymers (Basel). 2021 Jan 25;13(3):368. doi: 10.3390/polym13030368 (PMC7865594; doi:10.3390/polym13030368)
Supplement: Supplementary file 1 [file polymers-13-00368-s001.pdf]

Supplementary Information

Enhanced Electromechanical Property of Silicone Elastomer Composites Containing TiO_2@SiO_2 Core-Shell Nano-Architectures

Shuyan Gao [1], Hang Zhao [1,*], Na Zhang [1] and Jinbo Bai [2,*]

[1] State Key Laboratory of Photon-Technology in Western China Energy, and Institute of Photonics & Photon-Technology, Northwest University, Xi'an 710069, China; Gaoshuyann@126.com (S.G.); zhangna1@stumail.nwu.edu.cn (N.Z.)

[2] Laboratoire de Mécanique des Sols, Structures et Matériaux, CNRS UMR 8579, Centrale-Supélec, Université Paris-Saclay, 8-10 rue Joliot Curie, 91190 Gif-sur-Yvette, France

* Correspondence: hang.zhao@nwu.edu.cn (H.Z.); jinbo.bai@ecp.fr (J.B.)

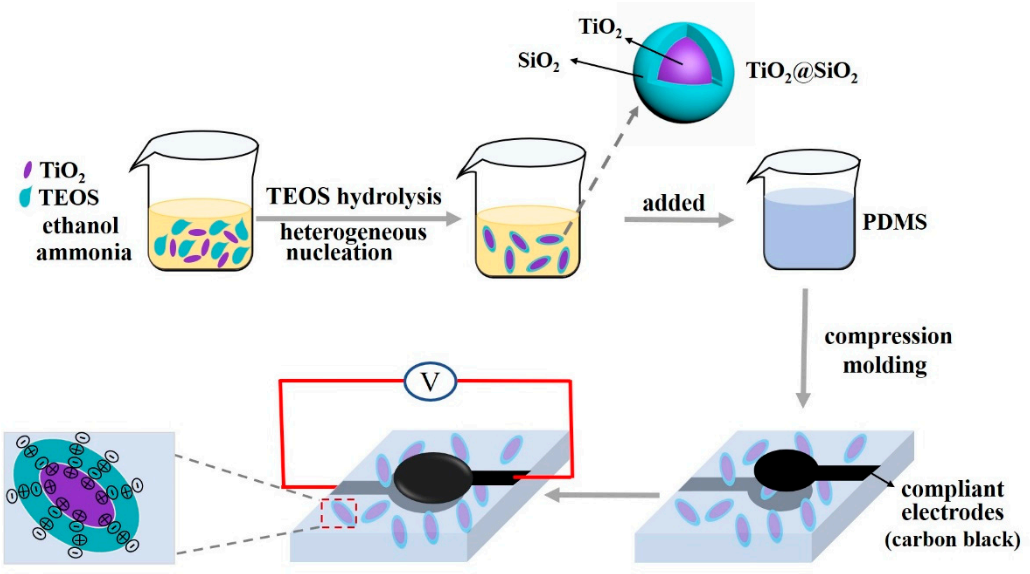

Figure S1. Schematic diagram of the preparation procedure of TiO_2@SiO_2 core-shell nanoparticles and composites.

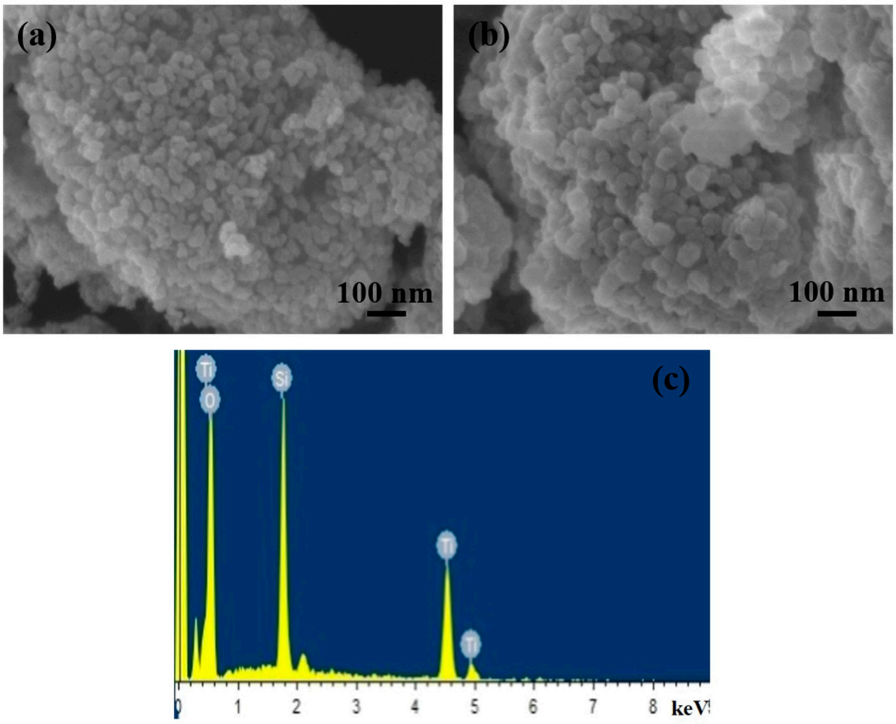

Figure S2. Scanning electron microscopy (SEM) images of (**a**) TiO2 and (**b**) TiO2@SiO2 nanoparticles; (**c**) energy-dispersive X-ray spectroscopy (EDS) image of TiO2@SiO2 nanoparticles.

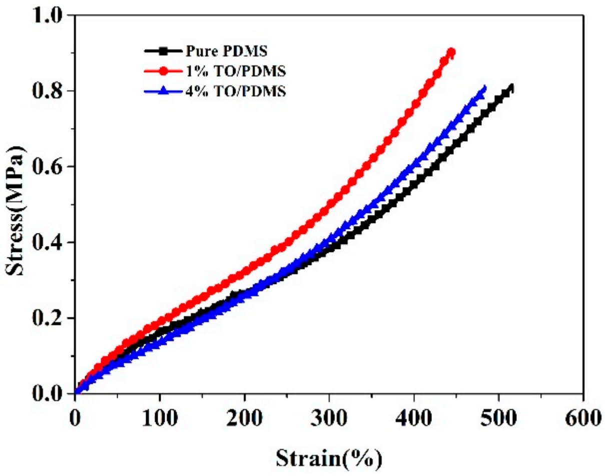

Figure S3. Stress-strain curves of TiO2/ polydimethylsiloxane (PDMS) composites.

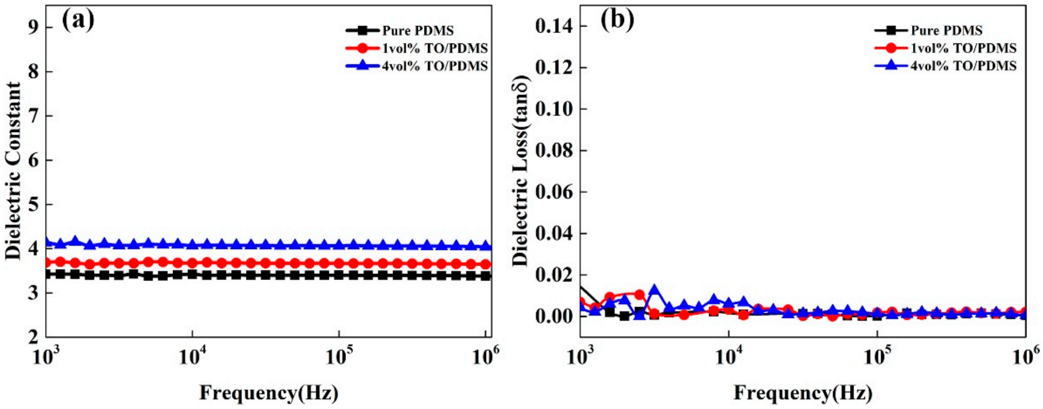

Figure S4. Frequency dependence of (**a**) dielectric constant, (**b**) dielectric loss in TiO2/PDMS composites.

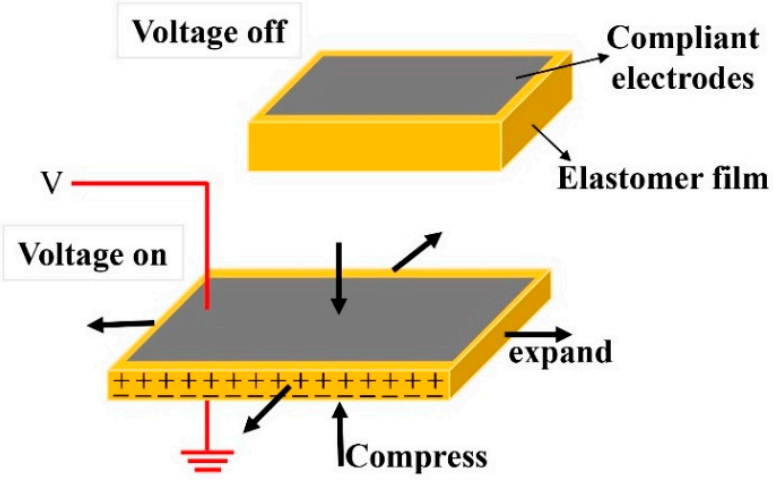

Figure S5. Operating principle of dielectric elastomer actuator.
